# Supplementary material for: Thirty Minutes Identified as the Threshold for Development of Pain in Low Back and Feet Regions, and Predictors of Intensity of Pain during 1-h Laboratory-Based Standing in Office Workers
Source: Int J Environ Res Public Health. 2022 Feb 16;19(4):2221. doi: 10.3390/ijerph19042221 (PMC8871560; doi:10.3390/ijerph19042221)
Supplement: Supplementary file 1 [file ijerph-19-02221-s001.zip › ijerph-1555146-supplementary.pdf]

a)

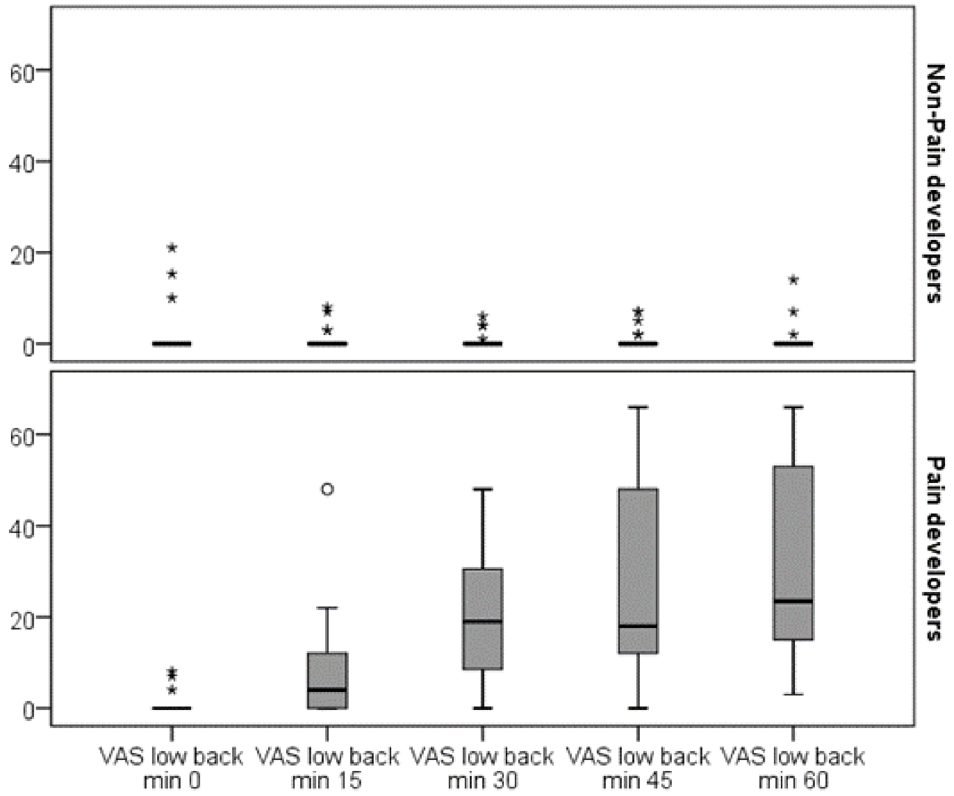


b)

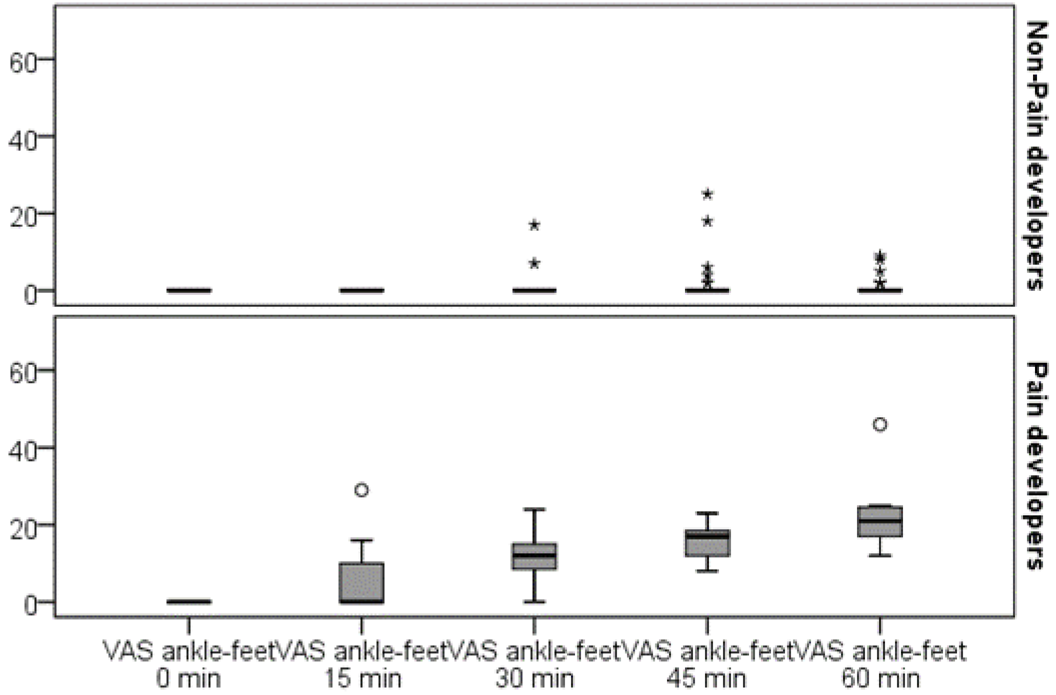


**Figure S1.** Pain development during 1h-standing for PD and NPD groups: (**a**) at low back; (**b**) at ankle-feet areas

**Table S1**. Spearman correlation coefficients between the maximum increment at VAS scores at low back and ankle-feet regions throughout of the standing task and measured variables for PD groups.

| | **Low back** | | **Ankle-feet** | |
|---|---|---|---|---|
| | PD (n = 14) | | PD (n = 9) | |
| | Rho | p | Rho | p |
| Age (years) | -0.082 | 0.780 | 0.083 | 0.831 |
| BMI (kg/m2) | 0.262 | 0.365 | 0.017 | 0.966 |
| IPAQ, during de last 7 days, how much time did you usually spend sitting on a weekend day (minutes) | 0.047 | 0.874 | -0.347 | 0.360 |
| IPAQ, during de last 7 days, how much time did you usually spend sitting on a weekday (min) | 0.544* | 0.044 | 0.525 | 0.146 |
| IPAQ, MET min/week | 0.020 | 0.946 | 0.583 | 0.099 |
| LBP severity, last 7 days (0-100) | 0.463 | 0.096 | 0.365 | 0.334 |
| OSPAQ, minutes sitting at work per week | -0.245 | 0.398 | -0.210 | 0.587 |
| OSPAQ, minutes standing at work per week | 0.049 | 0.869 | 0.619 | 0.075 |
| OSPAQ, minutes walking at work per week | 0.073 | 0.804 | -0.345 | 0.364 |
| JCQ, Job Control | 0.583* | 0.029 | -0.133 | 0.732 |
| JCQ, Psychological Job Demands | 0.392 | 0.165 | -0.099 | 0.800 |
| JCQ, Social Support | 0.323 | 0.260 | 0.365 | 0.334 |
| JCQ, Physical Demands | -0.395 | 0.162 | 0.070 | 0.859 |
| PCS, Rumination | 0.367 | 0.196 | 0.176 | 0.651 |
| PCS, Magnification | -0.242 | 0.404 | 0.121 | 0.756 |
| PCS, Helplessness | 0.164 | 0.574 | 0.305 | 0.425 |
| PCS-total | 0.142 | 0.627 | 0.117 | 0.765 |
| SF-12, Mental Component Summary | 0.037 | 0.899 | 0.117 | 0.765 |
| SF-12, Physical Component Summary | -0.522 | 0.056 | -0.083 | 0.831 |
| ASLR, total examiner-score (0-10) | 0.551* | 0.041 | 0.220 | 0.569 |
| ASLR, total participant-score (0-10) | 0.382 | 0.177 | 0.456 | 0.218 |
| AHAbd, right side, examiner-score (0-3) | -0.034 | 0.907 | 0.128 | 0.742 |
| AHAbd, left side, examiner-score (0-3) | 0.248 | 0.392 | 0.498 | 0.172 |
| AHAbd, right side, participant-score (0-5) | 0.217 | 0.457 | 0.492 | 0.179 |
| AHAbd, left side, participant-score (0-5) | 0.082 | 0.780 | 0.613 | 0.079 |
| Abdominal (s) | -0.198 | 0.497 | -0.433 | 0.244 |
| Side Bridge right side (s) | -0.578* | 0.030 | -0.533 | 0.139 |
| Side Bridge left side (s) | -0.383 | 0.176 | -0.267 | 0.488 |
| Supine Bridge (s) | -0.568* | 0.034 | 0.000 | 0.999 |
| Isometric hip abduction (right leg) (s) | -0.454 | 0.103 | -0.433 | 0.244 |
| Isometric hip abduction (left leg) (s) | -0.170 | 0.562 | -0.400 | 0.286 |
| Sorensen (s) | -0.289 | 0.317 | -0.967 * | 0.000 |

*$p \leq 0.05$; BMI, Body Mass Index; MET, Metabolic Equivalent of Task (computed as the sum of walking, moderate-intensity, and vigorous-intensity physical activity); OSPAQ, Occupational Sitting and Physical Activity Questionnaire; JCQ, Job Content Questionnaire; ASLR, Active Straight Leg Raise; AHAbd, Active Hip Abduction; s, seconds.
